# Simultaneous display of two large proteins on the head and tail of bacteriophage lambda

**DOI:** 10.1186/1472-6750-13-79

**Published:** 2013-09-30

**Authors:** Emiliano Pavoni, Paola Vaccaro, Valeria D’Alessio, Rita De Santis, Olga Minenkova

**Affiliations:** 1 Biotechnology, Sigma-tau, SpA, Pomezia (RM) 00040, Italy

**Keywords:** Lambda display, Nanoparticles, Tumor targeting, CEA

## Abstract

**Background:**

Consistent progress in the development of bacteriophage lambda display platform as an alternative to filamentous phage display system was achieved in the recent years. The lambda phage has been engineered to display efficiently multiple copies of peptides or even large protein domains providing a powerful tool for screening libraries of peptides, proteins and cDNA.

**Results:**

In the present work we describe an original method for dual display of large proteins on the surface of lambda particles. An anti-CEA single-chain antibody fragment and green fluorescent protein or alkaline phosphatase were simultaneously displayed by engineering both gpD and gpV lambda proteins.

**Conclusions:**

Here we show that such modified phage particles can be used for the detection of target molecules *in vitro* and *in vivo*. Dual expression of functional moieties on the surface of the lambda phage might open the way to generation of a new class of diagnostic and therapeutic targeted nanoparticles.

## Background

Bacteriophages, first discovered by Twort and D’Herelle at the beginning of the last century, are viruses that infect bacteria [1,2]. Because of their simple organization they became a favourite object in scientific research and for a long time their morphogenesis, infection mechanisms, assembly and genetics were studied providing the basis for many phage-derived techniques in molecular biology. One of the most popular phage-based technology is a phage display, introduced by Smith in 1985 [3]. First developed on filamentous single-stranded DNA bacteriophages fd, M13 or related phagemids, this technology is based on the insertion of foreign nucleotide sequences into genes encoding for various coat proteins resulting in a heterogeneous mixture of phages, each displaying the different peptide encoded by the corresponding insert. A physical link between a displayed fusion protein and the DNA encoding for it makes this phage target selectable, providing a powerful tool for a high throughput screening of large phage surface-displayed libraries of various origins.

Display of peptides or proteins on the surface of the filamentous phage is generally based on the fusion with the phage genes encoding for the coat proteins pVIII or pIII. It is possible to display a high number of short peptides fused to the small highly abundant major coat protein pVIII, which is particularly sensitive to the foreign peptide insert length [4]. In alternative, a very low number of larger polypeptides can be displayed by fusion to the tolerant minor protein pIII, which however, is only present in five copies at one end of the phage filament. In this latter case the avidity of the recombinant phage for its ligand is dramatically reduced, thus, seriously limiting the selection efficiency of ligands for receptors only available at low concentration or present in complex mixtures (as in the case of biological fluids, such as serum). Another factor that can influence the exposition of the foreign protein on the phage surface is connected with the viral life cycle. The filamentous phage assembly occurs within the bacterial membrane without causing cell lysis [5]. The capacity of a foreign protein to be secreted across the membrane depends on its amino acid composition [4,6-8], therefore filamentous phage-based libraries only display those recombinant proteins able to pass through the inner bacterial membrane still maintaining their correct folding in the oxidizing environment of the periplasmic space.

As an alternative, more recently developed phage display system, based on the lambda bacteriophage, overcomes most of these problems see for review [9]. Indeed, the lambda capsid is assembled in the cytoplasm of bacteria and mature phage particles are released by cell lysis. Thus, display of the fusion proteins on lambda phage does not depend on their ability of being translocated across the membrane. Moreover, lambda is a temperate phage, this means that the phage DNA can be inserted into the host genome, inducing lysogenic state of bacteria when expression of the phage proteins is completely suppressed, while the expression from a non-phage promoter controlling production of the fused proteins in the two gene-based lambda vectors remains at a very low levels, because of their low copy number in bacterial cell. For this reason, the display of some proteins interfering with bacterial vital functions and toxic for the host cell is feasible using lambda phage. Another advantage offered by lambda is its tolerance to the large polypeptide inserts in combination with a high density of their display. In fact, foreign proteins as large as scFv antibodies can reach 50% of the total gpD in case of expression as fusion at its N-terminus and up to 90% in case of C-terminal fusion [10].

The lambda displayed libraries proved to be very useful for particular applications, such as determination of the minimal binding or functional domain within a single gene product [11-14] or for screening with monoclonal antibodies cDNA libraries constructed from small viral genome [15]. Moreover, screening of large lambda libraries displaying complex protein repertoires derived from the cDNA of the tumor cells or tumor tissues with sera from breast cancer patients was shown to be an efficient method for the identification of novel tumor antigenic determinants [16-18].

The interest of scientists for the lambda phage with modified surface proteins is directed not only to the traditional use of phage libraries as a tool for molecular interaction studies, but also to possible medical or veterinary applications as efficient immunogen [19] or, possibly, as a potential delivery vehicle for gene therapy [20,21].

In the present work we describe a method for dual display of large proteins on the surface of the lambda particles. An anti-CEA single-chain antibody fragment and green fluorescent protein or alkaline phosphatase were simultaneously displayed by engineering both gpD and gpV lambda proteins. Here we show that such modified phage particles can be used for the detection of target molecules *in vitro* and *in vivo*. Dual expression of functional moieties on the surface of the lambda phage might open the way to generation of a new class of diagnostic and therapeutic targeted nanoparticles.

## Methods

### Bacterial and phage strains

*Escherichia coli* strain BB4 was used for phage plating and amplification (*supF*58 *supE*44 *hsdR*514 *galK*2 *galT*22 *trpR*55 *metB*1*tonA* Δ*lac*U169 F’[*proAB*^+^ lacI^q^*lacZ*ΔM15Tn10(*tet*^*r*^)]).

λKM8 and λKM10 are bacteriophage lambda display vectors [17] allowing to generate fusions with N- or C-termini of gpD, respectively. Both of these vectors are derivatives of λKM4 [16], obtained by introducing a flexible GS-linker between the displayed protein and gpD. These vectors are based on a double gene *D* system, where the lambda genomic copy of *gpD* gene harbors an amber mutation; the additional copy of *D* under the control of *Ptrc* promoter contains *Spe*I, *Not*I unique cloning sites situated at the 5′ or 3′ end of *gpD* gene. An ampicillin resistance gene allows propagation of the phage as Ap-resistant lysogenic colonies. Phage particles grown on suppressor bacterial strain display on their capsids a chimeric array of wild type gpD (encoded by the *gpD* lambda genomic gene) and recombinant gpD (encoded by the additional copy of gp*D* gene).

### Construction of lambda phages displaying GFP on N- and C-termini of gpD

The GFP gene was PCR amplified from pEGFP-N1 plasmid (Clontech) with two pairs of the primers KM491-KM492 and KM493-KM494 (Table 1) to clone GFP gene in λKM8 and λKM10 respectively. Both oligonucleotide pairs introduced either SpeI or NotI cloning sites (lowercase letters) and the pair KM493-KM494 introduced TAG and TAA codons (shown in bold) at the beginning and at the end of the amplified GFP gene, respectively. The amplified PCR products were purified, digested with SpeI and NotI, and ligated into the lambda vectors λKM8 and λKM10 [17], digested with SpeI and NotI, to obtain GFP-N-λ and GFP-C-λ, respectively.

**Table 1:** Oligonucleotide primers used in this study

**Name**	**Sequence (5′-->3′)**
KM491, FOR	(GTC)_4_actagtGTGAGCAAGGGCGAGGAGCTGTTC
KM492, REV	(GTC)_4_gcggccgcCCTTGTACAGCTCGTCCATGCCGA
KM493, FOR	(GTC)_4_actagt **TAG**GTGAGCAAGGGCGAGGAGCTGTTC
KM494, REV	(GTC)_4_gcggccgc**TTA**CTTGTA CAGCTCGTCCATGCCGA
KM549, FOR	**ATGCTGGACACCTCC** TGTGGTTCTGGATCAGGTTCTGGA *GTGAGCAAGGGCGAGGAG CTGTTCA*
KM550, REV	**AACCAGCTTACGGCT** ACATCCAGAACCTGATCCAGAACC *CTTGTACAGCTCGTCCATG CCGAG*
KM553, FOR	**GCGTGGGATGGCACC** TGTGGTTCTGGATCAGGTTCTGGA *GTGAGCAAGGGCGAGG AGCTGTTC*
KM554, REV	**GGCAGCACCGTCGGT** ACATCCAGAACCTGATCCAGAACC *CTTGTACAGCTCGTCCAT GCCGAG*
KM555, FOR	**GCCAGCGACGAGACG** TGTGGTTCTGGATCAGGTTCTGGA *TGAGCAAGGGCGAGGAG CTGTTCA*
KM556, REV	**AAACGCGGTCCGTTTTTT** ACATCCAGAACCTGATCCAGAACC *TTGTACAGCTCGTCCAT GCCGAG*
K47, FOR	GGGCACTCGACCGGAATTATCG
KM541, REV	*CTCGCCCTTGCTCAC* TCCAGAACCTGATCCAGAACCACA **GGAGGTGTCCAGCATCA GCGGGGT**
KM543, REV	*CTCGCCCTTGCTCAC* TCCAGAACCTGATCCAGAACCACA **GGTGCCATCCCACGCAA CCAGCTT**
KM544, REV	*CTCGCCCTTGCTCAC* TCCAGAACCTGATCCAGAACCACA **CGTCTCGTCGCTGGCAG CCTCCGG**
KM545, FOR	*GACGAGCTGTACAAG* GGTTCTGGATCAGGTTCTGGATGT **AGCCGTAAGCTGGTTGCGT GGGAT**
KM547, FOR	*GACGAGCTGTACAAG* GGTTCTGGATCAGGTTCTGGATGT **ACCGACGGTGCTGCCG TTGGCA**
KM548, FOR	*GACGAGCTGTACAAG* GGTTCTGGATCAGGTTCTGGATGT **AAAAAACGGACCGCGTTT GCCGGA**
KM60, REV	GATTTAATCTGTATCAGGCTGA
KM 513, REV	TACGGCTGGAG GTGTCCAGCATCAG
K86, REV	CTCTCATCCGCCAAAACAGCC
KM512, FOR	CTGATGCTGGACACCTCCAGCCGTA
KM526, FOR	GTCGTCGTCgcggccgcA**AGGAGGA**AATCCTCATGCCTGTACCAAATCCTACAATG
KM527, REV	CCCTTTCACCACCGA GGTGCTG
KM530, FOR	GAGGTGCAGCTGTTGGAGTCT
K48, REV	GGTATGAGCCGGGTCACTGTTG
KM215	TCAGGTGGAGGCGGTTCAGGCGGAGGTGGCTCTGGCGGTGGCGGATCG
KM528, FOR	AGCACCTCGGTGGTGAAAGGGTCAGGTGGA
KM529, REV	CTCCAACAGCTGCACC TCCGATCCGCCACC
SM132, FOR	GTCGTCGTCactagt*TAG***TCAGGCGGTGGATCAGGCGGTGGATCAGGCGGTGGATCA**CGGACACCAGAAATGCCTGTTCTG
SM133, REV	GTCGTCGTCctgcag*TTATTTCAGCCCCAGAGCGGCTTTCATG*
SM134, FOR	GTCGTCGTCctgcagA**AGGAGGA**AATCCTCATGCCTGTA

### Construction of lambda phages displaying GFP in different positions of gpD

The GFP gene was PCR amplified from pEGFP-N1 plasmid (Clontech) with three pairs of the primers KM549-KM550, KM553-KM554, KM555-KM556 (Table 1). The 3′-ends of the primers (in italic) are complimentary to the *GFP* gene, the central part in each oligonucleotide encodes for C(GS)_3_G linker sequence and the 5′-ends (in bold) are complimentary to various regions of the *gpD* gene, thus allowing the assembly of GFP-gpD fusion proteins, where GFP is inserted between 42–43 or 52–53, or 95–96 amino acids of gpD, respectively (not counting the initial methionine, which is not present in the mature protein). The *gpD* gene fragments were amplified with following primers: 1–43 aa (K47-KM541), 44–110 aa (KM545-KM60), 1–53 aa (K47-KM543), 54–110 aa (KM547-KM60), 1–96 aa (K47-KM544), 97–110 aa (KM548-KM60). The 3′-ends of the primers (in bold) are complimentary to the corresponding regions of the *gpD* gene, the central part in each oligonucleotide encodes for C(GS)_3_G linker sequence and the 5′-ends (in italic) are complimentary to the *GFP* gene. External primers K47 and KM60 were positioned upstream and downstream of *gpD*, respectively. The overlapping fragments were assembled in the unique gene encoding for the gpD-GFP-gpD by 20 cycles of PCR-like amplification without primers as it is generally used for scFv gene assembly [22]. K47 and KM60 external primers were then added and the reaction was cycled another 25 times. PCR product was gel purified, digested with NcoI and EcoRI, and ligated into the plasmid of pKM4 [16], digested with NcoI and EcoRI. The resulting plasmid was XbaI–linearized and inserted into the XbaI (24508) site of the lambda.

### Construction of lambda phage displaying anti-CEA scFv antibody at the N-terminus and GFP at the C-terminus of gpD

The anti-CEA scFv antibody-encoding DNA fragment was PCR amplified by using as template the α-CEA-N-λ phage [10]. The forward primer K47 and reverse primer KM513 were used. The resulting DNA fragment contained encoding sequence for the anti-CEA scFv and the N-terminal half of gpD.

The GFP gene was PCR amplified by using as template the GFP-C-λ phage, described in this study. The forward KM512 and reverse primers KM86 were used. The resulting DNA fragment encoded for C-terminal half of gpD and the GFP, overlapping with previously obtained fragment. The fragments *scFv-gpD* and *gpD-GFP* were assembled in the unique gene encoding for the scFv-gpD-GFP by 20 cycles of PCR-like amplification without primers. K47 and K86 external primers were then added and the reaction was cycled another 25 times. PCR product was gel-purified, digested with NcoI and EcoRI, and ligated into the plasmid of pKM4 [16], digested with NcoI and EcoRI. The resulting plasmid was XbaI–linearized and inserted into the XbaI (24508) site of lambda.

### Construction of lambda phage displaying anti-CEA scFv antibody on the tail protein gpV and GFP on the head protein gpD

Lambda phage with simultaneously modified gpD and gpV proteins was constructed from GFP-C-λ phage, described in this study. First, the truncated gpV gene was PCR amplified from the phage genome using the primers KM526 and KM527 (the NotI restriction site in KM526 is shown in lowercase letters, Shine-Dalgarno sequence is shown in bold). Second, the anti-CEA scFv gene was amplified from α-CEA-C-λ [10] with the primer KM530 and a downstream primer K48. A DNA fragment encoding for the linker sequence S(GGGGS)_3_ and flanked with the short complimentary sequences to the truncated gpV and anti-CEA scFv genes, at its 3′ and 5′ ends respectively, was obtained by PCR amplification of template KM215 with the primers KM528 and KM529.

These three fragments were purified by using the PCR purification kit (Qiagen, MD, USA) and assembled in unique gene encoding for the gpV-linker-scFv by 20 cycles of PCR-like amplification without primers. The external primers KM526 and K48 were then added to the mixture and the reaction was cycled another 25 times. PCR product was gel purified, digested with NotI and ligated into the GFP-C−λ phage, digested with NotI.

### Construction of lambda phage displaying anti-CEA scFv antibody on the tail protein gpV and alkaline phosphatase on the head protein gpD

First, alkaline phosphatase gene (*PhoA*) was PCR amplified from *E. coli* genome using the primers SM132 and SM133. The 3′-ends of the primers (in italic) were complimentary to the *PhoA*. The central part of the SM132 primer encoded for the (SGGG)_3_S linker (in bold) and contained an *amber* codon (in italic) and SpeI restriction site (lowercase letters). The SM133 contained PstI restriction site (lowercase letters). Second, the gene encoding for gpV-linker-scFv was amplified from phage GFP/α-CEA-λ, obtained in this study, by using the forward primer SM134 and reverse external primer KM60. The SM134 contained PstI restriction site (lowercase letters), a Shine-Dalgarno sequence (in bold) and ATG codon. Then, the DNA fragments were purified, digested with PstI restrictase and ligated. The resulting DNA fragment was purified from agarose gel, then digested with SpeI, NotI restrictases and cloned in λKM10, digested with SpeI and NotI.

### Evaluation of recombinant protein loading in GFP-N-λ and GFP-C-λ phages

The GFP-N-λ and GFP-C-λ phages were initially purified by PEG and NaCl precipitation, followed by centrifugation in CsCl gradient [23]. The about 10^8^ PFUs of the purified GFP-C-λ phage were fractionated by SDS/PAGE, transferred onto a nitrocellulose membrane (BioTrace NT, Pall, Gast Hills, NY), probed with rabbit anti-D polyclonal serum (dilution 1:150) and developed with an AP-conjugated gamma chain specific anti-rabbit monoclonal antibody (Sigma A 2556, dilution 1:5000) in order to identify wild type and recombinant gpD positions on the membrane. Along with that about 10^9^ PFUs of the GFP-C-λ were fractionated by SDS/PAGE and stained with Coomassie Blue. Then relative abundance of GFP-gpD fusion protein as compared to wild type gpD on lambda phage capsid migrating in the expected positions were estimated by densitometric scanning of the stained gel, performed with STORM 840 (Molecular Dynamics, Amersham). The resulting image was integrated by using Image Quant 5.1 software. Then, serial dilutions of the GFP-N-λ and GFP-C-λ phages were blotted onto nitrocellulose membrane and developed with an anti/GFP antibody to estimate relative loading of GFP on the capsid of the GFP-N-λ phage.

### Lambda ELISA

ELISA against CEA using phage lysate was performed as previously described [16]. Briefly, multi-well plates (Nunc-Immuno Plate, Nunc, Denmark) were coated overnight at 4°C with 100 μl/well of 10 μg/ml CEA protein, purified from human fluids (TriChem, West Chester, PA), in coating buffer (50 mM NaHCO_3_, pH 9.6) or GST as negative control. Anti-CEA scFv bound lambda phage was revealed using a polyclonal anti-lambda phage rabbit antibody in concentration 0.64 μg/ml, followed by incubation with AP or HRP conjugated anti-rabbit secondary antibody (Sigma A-2556 or Sigma A-1949, respectively).

ELISA to normalize the number of physical particles in the phage samples were done by using ELISA plate coated with 100 μl of 10 μg/ml of anti-gpV monoclonal antibody. Then known amounts of PFUs of the phage were added to the wells and after incubation the bound phage was revealed as above.

For evaluation of the display efficiency of the GFP on recombinant phages, an anti-GFP goat polyclonal AP-conjugated antibody (GTX26661, GeneTex. Inc) was used.

### Phage targeting *in vitro*

LoVo cells were plated in 6-well plate (Biocoat, PA, USA) at a concentration of 100.000 cells/cm^2^. Next day the cells were washed four times with 2 ml of PBS and blocked by adding 5% skim milk in PBS for 1 h at RT. After discarding the blocking solution, one ml of blocking solution containing about 10^10^ phage particle was added to the cells and incubated for 1 h at 37°C. The wells were washed for 4 times with blocking solution and the bound phage was eluted with 400 ml of 0.1 M glycine, pH 2.2, neutralized and assayed by using GFP ELISA kit (cat. AKR-121, Cell Biolabs).

### Phage targeting *in vivo*

Nude mice (n.36) were grafted subcutaneously with two human carcinoma cell lines previously shown to express CEA. About 2.5×10^6^ HT29 human colon carcinoma cells, were injected in the right flank of the mice and 5×10^6^ LoVo human colorectal carcinoma cells in the left flank. When tumor masses reached 100–300 mg, the mice were divided into three groups and injected intravenously with 10^10^ phage particles of λKM8, α-CEA-N-λ, or GFP/α-CEA-λ phage. After 24, 48 and 72 h four mice of each group were sacrificed and blood, liver, spleen, brain, muscle and tumor samples collected. All the samples were weighed and homogenized in 1 mL of sterile PBS. Different dilutions of these homogenates containing lambda phage were tested on BB4 lawn and PFU counted. Several tumor samples were divided into two parts and second piece was immersed in OCT compound (36160 3E, BDH, England) and frozen for further immunohistochemistry analysis.

The samples cryoprotected in OCT were sectioned frozen on a cryostat at 15 μm thickness and the slides stored at −80°C. The sections were postfixed with acetone for 30 min and rinsed with PBS three times for 5 min. Subsequently the sections were incubated overnight with the primary anti-lambda polyclonal rabbit antibody diluted 1: 1000. Then, the section were rinsed three time in PBS and incubated with the secondary Cy2 anti-rabbit antibody (Jackson Immunoresearch) for 2 h at room temperature. Sections were rinsed three times for 15 min in 0.1 M phosphate buffer and mounted on gelatin-coated slides. Images were acquired by using a Zeiss LSM 700 laser-scanning confocal microscope under nonsaturating exposure conditions and using the same acquisition settings for all groups.

All studies involving animals were conducted in accordance with European Directives no. 86/609, Italian Legislation D.L. 116, January 27th, 1992 and ARRIVE guidelines {Kilkenny, 2012 461 /id}. The protocol B-IMM-11-10 applied in this study was approved by veterinary of Sigma-tau, SpA and authorized by decree of the Ministry of Health of Italy (decree number: 12/2010-B, January 25, 2010).

### Immunoscreening with anti-GFP antibody

To analyse the GFP expression stability the filters with blotted phage plaques were reacted and developed with an anti-GFP AP-conjugated antibody (Gtx26661, GeneTex, CA, diluted 1:1000).

## Results

### Display of GFP as N-terminal or C-terminal fusion

**Figure 1:**
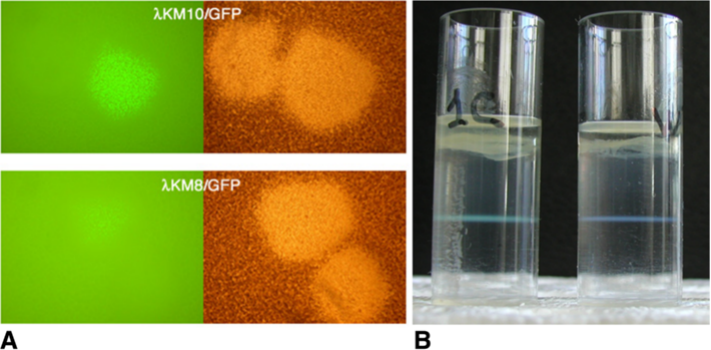
**Construction of phages displaying GFP on their surface. A**. Fluorescent plaques were observed after cloning of the GFP in the λKM8 (top picture) and λKM10 (lower picture) allowing to generate fusions to the N- and C- termini of the gpD, respectively, and to display GFP on the lambda capsid. Empty clones after cloning were not fluorescent. The obtained phages were named GFP-N-λ and GFP-C-λ. **B**. Purification of GFP-C-λ (left tube) and wild type lambda (right tube) in CsCl gradient.

We previously showed that a protein large as scFv antibody (242 aa) can be efficiently displayed on the lambda capsid in functional form as N- or C-terminal fusion to the gpD head protein [10]. The density of the recombinant fusion proteins on the lambda capsid in the two gene-based system was about 50% and 90% of total gpD for N- and C-terminal fusions, respectively. However, the phage with the C-terminal fusion was less productive, forming fewer and smaller plaques. This recombinant phage was also found to accumulate nonsense mutations, such as stop codons or frame shifts, that are able to block the fusion protein expression resulting in better phage particle production. In this study the reporter gene encoding the GFP (238 aa) was cloned into λKM8 and λKM10 vectors to get N- and C- terminal fusions of the gpD, respectively, as described in Methods. In order to improve stability and viability of the GFP C-terminal fusion bearing clone we introduced an amber codon for the conditional expression of the fusion protein through a host bacteria suppressor activity in BB4 bacterial stain. The ligated DNAs after packaging were plated in NZY-top agar and directly observed in fluorescent microscope with the minimal magnification ×10 (Figure 1A). The clones encoding for the fused GFP were easily identified under the microscope. They were named GFP-N-λ and GFP-C-λ according to the fusion site in gpD. The phage with the C-terminal fusion, GFP-C-λ, formed more intense fluorescent plaques. Furthermore, in the presence of amber codon between gpD and GFP genes this phage formed normal-size plaques and stably expressed GFP even after several cycles of phage amplification. We measured the incorporation efficiency in bacteriophage lambda capsid of GFP fused to either terminus of gpD. The amount of GFP incorporated into lambda phage capsid depended on the insertion site. In case of the C-terminal fusion, 30% of total gpD protein was found to be fused to GFP (data not shown), while in the GFP-N-λ phage the portion of the recombinant gpD was about 15%. The GFP-C-λ phage particles, purified in CsCl gradient, were clearly visible as a green colored band in the daylight (Figure 1B).

### Lambda phages displaying GFP in different positions of gpD

**Figure 2:**
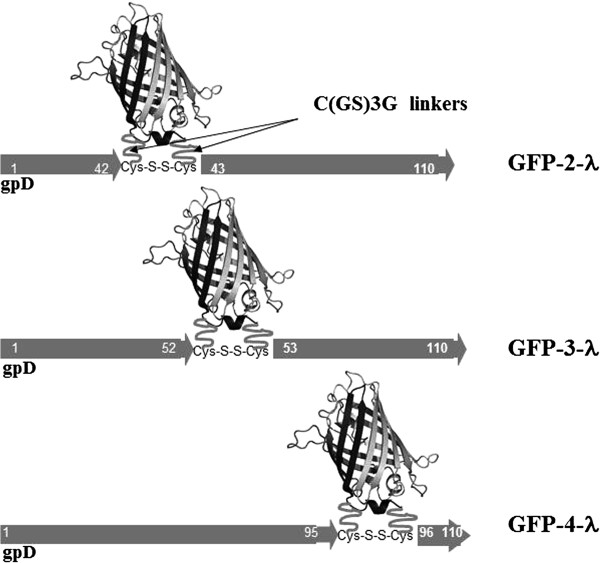
Schematic representation of recombinant gpD proteins fused to the GFP in the phages GFP-2-λ, GFP-3-λ, GFP-4-λ.

Both termini of gpD are located on the capsid-proximal side of the gpD trimer and faced to the virion according to crystal structure of gpD obtained by cryo-electron microscopy [24]. Therefore, theoretically, proteins fused to either terminus of gpD should not be exposed on the phage surface. Nevertheless, both N- and C-terminal fusions have been successfully displayed and recombinant proteins were efficiently incorporated in the phage capsid previously [15,16,25-29]. Based on the study of Pluckthun [24], we analyzed the gpD structure and identified three new possible insertion sites in gpD located on the surface-exposed region of gpD, namely between Ser^42^-Ser^43^ (2^nd^ insertion site, considering the N-terminus as a first one), Tre^52^-Tre^53^ (3^rd^ site), and Tre^95^-Lys^96^ (4^th^ site). We cloned GFP in all the three sites adding C(GS)_3_G linkers on both sides of GFP insert to facilitate assembly of the large protein domain on the virion surface and outside cystein residues so as to stabilize phage particle, assuming that cystein residues will form disulfide bonds after oxidation of sulfhydryl groups under the storage (Figure 2).

**Figure 3:**
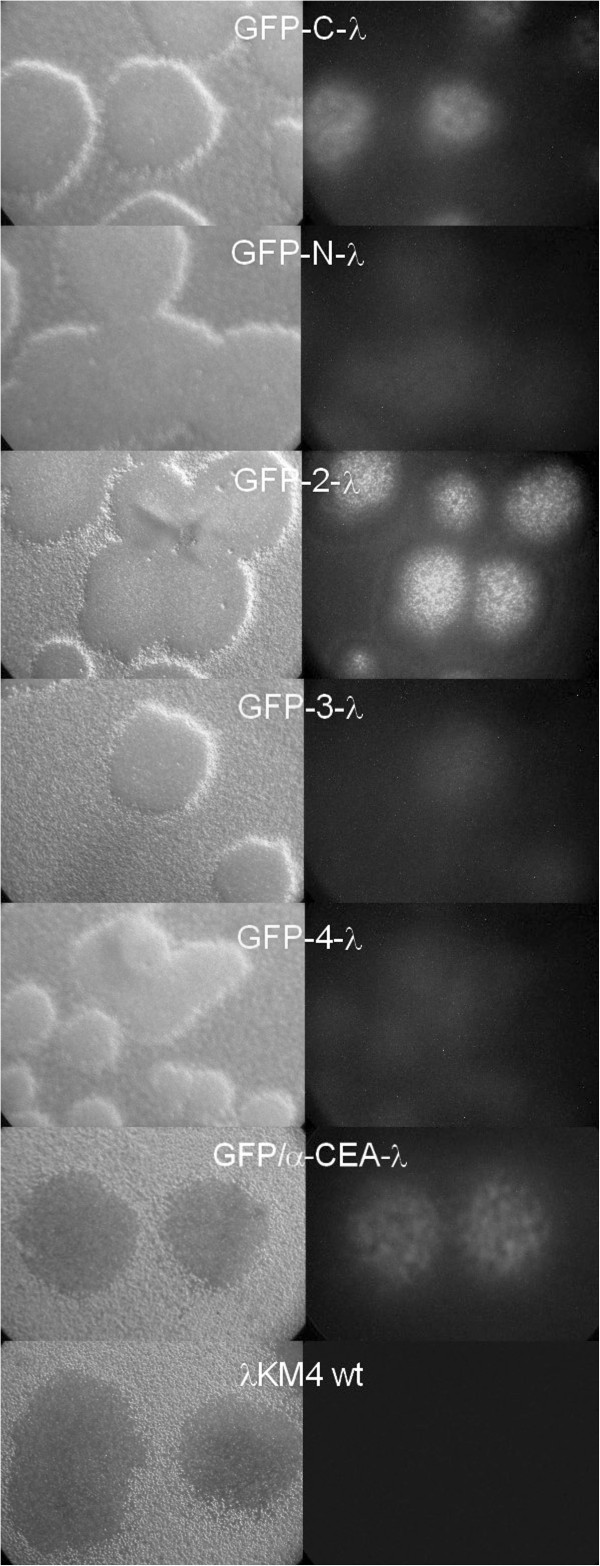
**Microscope observation of the lambda phage plaques displaying GFP as a fusion to the different sites of the gpD protein.** Lambda phage clones displaying GFP as C-terminal (GFP-C-λ), N-terminal (GFP-N-λ) fusion, fused to the new positions inside of the gpD protein (GFP-2-λ, GFP-3-λ, GFP-4-λ) or double displaying GFP/α-CEA-λ, were observed in fluorescent and light microscope. λKM4 wild type phage is included in the analysis as a negative control.

The phages displaying GFP in 5 different positions (N-terminus, 2, 3, 4, C-terminus) were observed by fluorescent microscope (Figure 3). The GFP expressed at the 3^rd^ and 4^th^ position formed fluorescent plaques of very low intensity similar to the GFP-N-λ (estimated by eyes). The phage with insert in the 2^nd^ position formed unexpectedly bright plaques, but sequence analysis showed the presence of the nonsense codon immediately after GFP gene, therefore this phage produced free GFP non exposed on the phage capsid. This phage was used as a negative control in further experiments. The cloning of the GFP gene in different gpD position was performed in two steps as described in Methods. First, we assembled GFP-gpD fused gene, cloned it in a plasmid and the correct sequences of the fused genes were confirmed by sequence analysis. Then the plasmids were inserted in the lambda genome. The positive recombinant lambda phages were isolated based on their fluorescence and sequenced again. Probably in the case of GFP-2-λ the correct plaques were hardly visible and a revertant clone with nonsense mutation was chosen.

**Figure 4:**
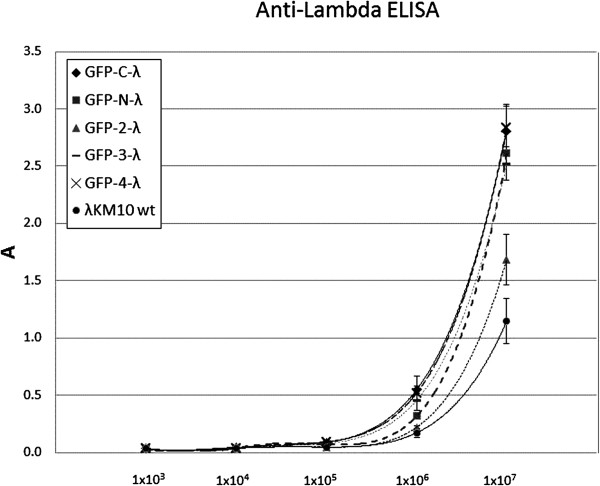
**Interference of GFP incorporation in lambda capsid with phage infectivity.** Lambda phages displaying GFP at the N-terminal end of the gpD (GFP-N-λ), at the C-terminal end (GFP-C-λ) or in new positions inside of the gpD protein (GFP-2-λ, GFP-3-λ, GFP-4-λ), were assayed by using anti-gpV antibody-coated ELISA plate and polyclonal anti-λ rabbit antibodies to reveal the bound phage. Phage quantities/well are indicated. Results are expressed as A=A_450_-A_620_. Data reported are the average values of assays performed in duplicate. Error bars represent the standard deviation of the mean.

To check whether the incorporation of the GFP into the phage capsid might interfere with phage infectivity, we measured PFUs in the fresh phage preparations and then analyzed the number of phage particles by ELISA, loading the same quantity of PFU per well of ELISA plate for all phages (Figure 4). Anti-gpV mouse mAb was used for coating ELISA plate and polyclonal anti-λ rabbit serum was applied for the bound phage detection. ELISA signals, proportional to the number of phage particles bound to the well, were found higher for phages GFP-N-λ GFP-3-λ GFP-4-λ GFP-C-λ as compared with wt phage. This means the infectivity of wt phage is 2–3 times higher than infectivity of GFP-phages. The infectivity of the GFP-2-λ, the phage harboring stop codon inside of the fusion gene was closer to wild type phage as this phage failed to incorporate GFP in the capsid.

**Figure 5:**
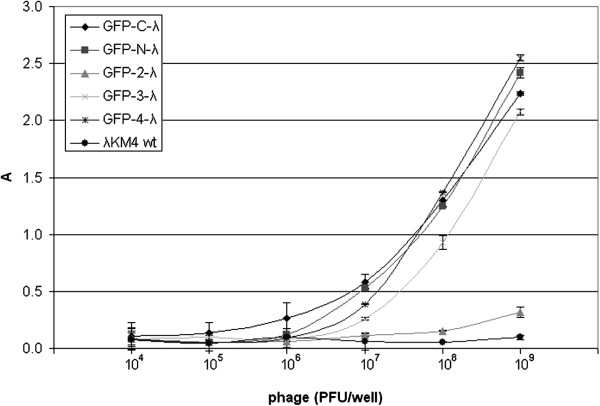
**Display efficiency of GFP fused to the different sites of the gpD head protein in lambda.** Lambda phage clones displaying GFP as N-terminal (GFP-N-λ), C-terminal (GFP-C-λ) fusions or fused to the new positions inside of the gpD protein (GFP-2-λ, GFP-3-λ, GFP-4-λ), were tested by ELISA using anti-GFP antibody. λKM4 wild type phage was tested as a negative control. On the abscissa are indicated the amount of wild type phage measured in pfu. The amounts of all other phages used in ELISA have been normalized relative to wild type phage, so as to have identical number of physical particles per well. ELISA was performed by using anti-gpV antibody-coated plates and the displayed GFP was revealed by using an anti-GFP AP-conjugated antibody. Results are expressed as A=A_405_-A_620_. Data reported are the average values of assays performed in duplicate. Error bars represent the standard deviation of the mean.

In further experiments the normalized amount of phage was applied on the anti-gpV-coated ELISA plate and then binding phage was detected with an anti-GFP antibody so as to see efficiency of GFP incorporation in different phages (Figure 5). In this assay, the capturing of phage particles with the anti-gpV mAb allowed an effective elimination of free recombinant GFP-gpD, which might be co-purified from supernatant by PEG precipitation together with bacteriophage particles. Similar signals with anti-GFP antibody, obtained for all phages, indicated that fused GFP-gpD protein can be efficiently incorporated in the capsid independently on the site of insertion. It also indicated that the incorporated GPF is exposed on the surface and available for anti-GFP antibody recognition. The brightness of GFP-phage plaques indicated the C-terminus as a better insertion site in terms of either phage particle yield or orientation of GFP on the capsid.

### Simultaneous display of the anti-CEA scFv antibody and GFP on the lambda capsid

At first, we tried to construct a double displaying bacteriophage expressing an anti-CEA scFv antibody as the N-terminal and GFP as the conditional C-terminal fusion to the gpD protein obtaining a very low percent of fluorescent plaques after plating. From 16 isolated fluorescent clones, only one clone contained both scFv and GFP genes, but already after a single amplification round this clone lost fluorescence while still displaying the scFv in a functional form (data not shown), thus leading us to the conclusion that the phage displaying two proteins fused to the same molecule of the head protein gpD is not stable.

**Figure 6:**
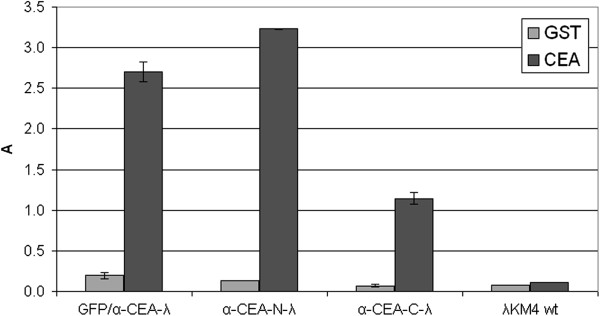
**Anti-CEA reactivity of the phage clones displaying anti-CEA scFv and GFP.** Lambda phage, displaying simultaneously GFP at the C-terminus of gpD and anti-CEA scFv antibody as fusion to the C-terminus of the truncated gpV tail protein (GFP/α-CEA-λ) was tested by ELISA against purified CEA and an irrelevant control protein (GST). Wild type phage λKM4 was tested as a negative control. The phage α-CEA-N-λ and α-CEA-C-λ displaying scFv antibody at the N- or C-terminus of the gpD respectively, were included as positive controls. The bound phage was detected by using rabbit anti-lambda polyclonal antibody. Results are expressed as A=A_450_-A_620_. Data reported are the average values of assays performed in duplicate. Error bars represent the standard deviation of the mean.

Therefore, we tested the simultaneous co-displaying of the GFP and scFv proteins, on the head and tail of lambda phage particles. It was shown earlier that the major tail protein gpV, with a 70 aa deletion on its C-terminus, is a suitable platform for the display of peptides and proteins [30]. In particular, we cloned the GFP at the C-terminus of gpD and the anti-CEA scFv at the C-terminus of a truncated version of gpV, thus, obtaining GFP/α-CEA-λ phage. This phage formed fluorescent plaques with brightness similar to GFP-C-λ (Figure 3) and efficiently bound CEA protein (Figure 6).

**Figure 7:**
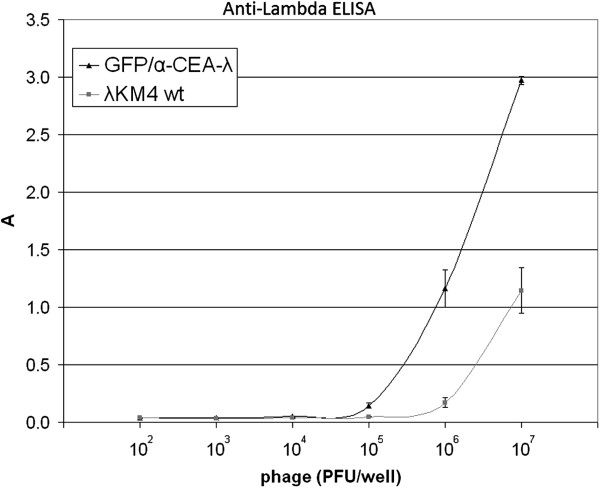
**Interference of GFP and scFv incorporation in the capsid of GFP/α-CEA-λ with phage infectivity.** Lambda double displaying phage GFP/α-CEA-λ and wild type λKM4 were assayed by using anti-gpV antibody-coated ELISA plate and polyclonal anti-λ rabbit antibodies to reveal the bound phages. The amounts of phage measured as PFUs used per well of ELISA plate, are indicated on the abscissa. Results are expressed as A=A_450_-A_620_. Data reported are the average values of assays performed in duplicate. Error bars represent the standard deviation of the mean.

**Figure 8:**
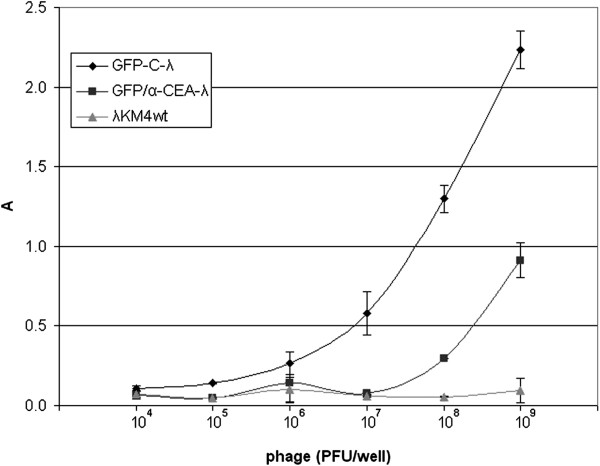
**Efficiency of GFP display on the capsid of the double displaying phage GFP/α-CEA-λ.** Lambda phage clones, displaying GFP at the C-terminus of gpD or double displaying GFP at the same position and anti-CEA scFv antibody as a fusion to the truncated gpV, were evaluated for the GFP display efficiency. On the abscissa are indicated the amount of wild type phage measured in pfu. The amounts of the phage used in ELISA have been normalized relative to wild type phage in order to have identical number of physical particles per well. ELISA was performed by using anti-gpV antibody-coated plates and the displayed GFP was revealed by using an anti-GFP AP-conjugated antibody. Results are expressed as A=A_405_-A_620_. Data reported are the average values of assays performed in duplicate. Error bars represent the standard deviation of the mean.

The ratio between phage particle number and PFU was about 10 times higher for GFP/α-CEA-λ phage than for wt phage (Figure 7). Also we observed a lower incorporation level of GFP in the lambda head for GFP/α-CEA-λ phage, as compared to the GFP-C-λ displaying GFP alone (Figure 8). We suppose that only a portion of GFP is incorporated in phage particle. This would explain the similar brightness of GFP/α-CEA-λ and GFP-C-λ plaques and different incorporation level.

### Targeting tumor cells *in vitro* and *in vivo*

**Figure 9:**
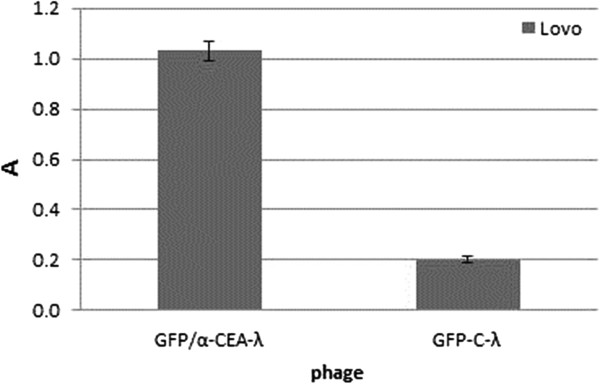
***In vitro *tumor cell targeting. GFP targeting to tumor cells by the double displaying phage GFP/α-CEA-λ was detected by ELISA on LoVo cells.** GFP was revealed by GFP ELISA kit. Results are expressed as A=A_450_-A_620_. Data reported are the average values of assays performed in duplicate. Error bars represent the standard deviation of the mean.

We tested the double displaying phage for binding to CEA-expressing tumor cells. LoVo colorectal carcinoma cells, known for their high expression of the CEA protein, were targeted by the phage particles. After incubation of live cells with the GFP/α-CEA-λ and GFP-C-λ phages, the cell bound phage was eluted by 0.1 M glycine, pH 2.2 and the GFP detected by using a GFP ELISA kit (Figure 9). The phage GFP/α-CEA-λ, displaying both targeting and detecting moieties, bound the CEA-expressing tumor cells selectively as compared to the phage expressing the GFP alone.

**Figure 10:**
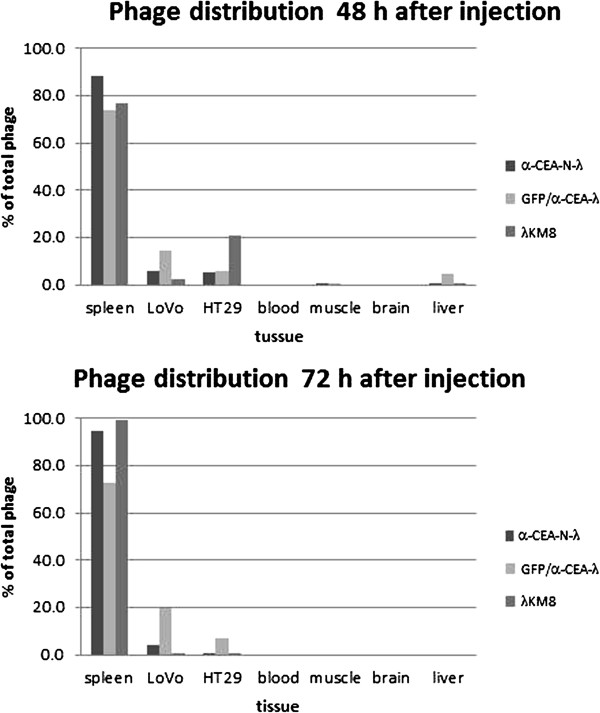
**Bacteriophage lambda distribution in mouse organs 48 and 72 h post phage i.v. injection.** Data expressed as a percent of the total infective phage particles, still remaining in mouse organs, 2 or 3 days after phage i.v. injection. Data reported are the average titers obtained from 4 animals.

**Figure 11:**
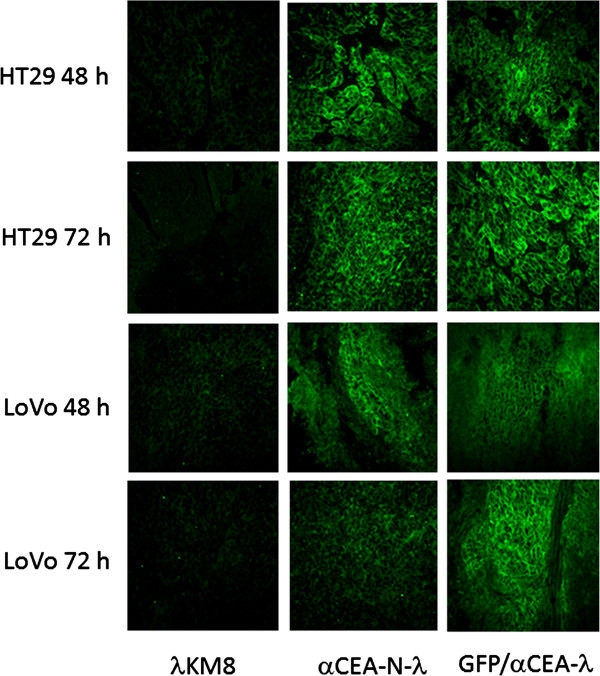
**Confocal microscopy of tumor bound phage.** Tumor bound phages were detected by staining with α-phage lambda polyclonal rabbit serum, followed by an anti-rabbit FITC-conjugated secondary antibody.

Then, the targeting moiety of the α-CEA-displaying phage was challenged *in vivo*. Nude mice were grafted subcutaneously with LoVo and HT29 cells. When tumor masses reached 100–300 mg, the mice were i.v. injected with the following bacteriophages: wild type phage λKM8; α-CEA-N-λ; GFP/α-CEA-λ. After 24, 48 and 72 h the mice were sacrificed and phage in different organs was counted. We found that phage titers decreased rapidly in all organs and tissues of the mice. After 24 h total phage quantity decreases by about 2 to 3 orders of magnitude and after 48 h about 4 orders of magnitude from the originally injected phage. Most of the remaining phage is localized in the spleen. We found that wild type lambda as well as the modified bacteriophages are accumulated in the spleen after i.v. or i.p. injection. Probably this can explain the efficiency of the lambda-based vaccines described in the literature [31,32]. However, after 72 h up to 20% of the remaining phage is detected in the tumor (Figure 10) when all other organs and tissues tested, apart of the spleen, are free of phage. Alternatively, the samples were frozen in OCT for the subsequent tissue staining. Immunofluorescent staining also confirmed phage localization in the tumor expressing CEA protein (Figure 11). Only tumors extracted from the mice treated with the anti-CEA targeting phages are stained with anti-lambda antibody.

### Simultaneous display of the anti-CEA scFv antibody and alkaline phosphatase (AP) on the lambda capsid

**Figure 12:**
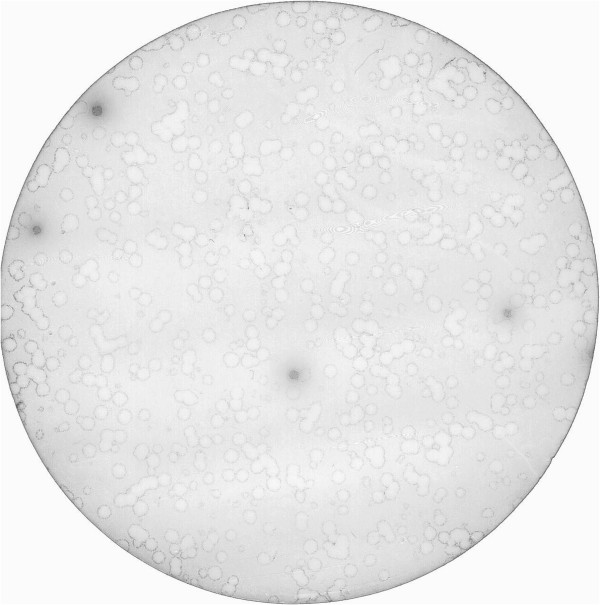
**Identification of phage clones displaying AP on the capsid.** The phage plaques after cloning the AP gene were transferred onto the nitrocellulose membrane. The positive clones were identified by incubating the membrane with an AP-substrate.

**Figure 13:**
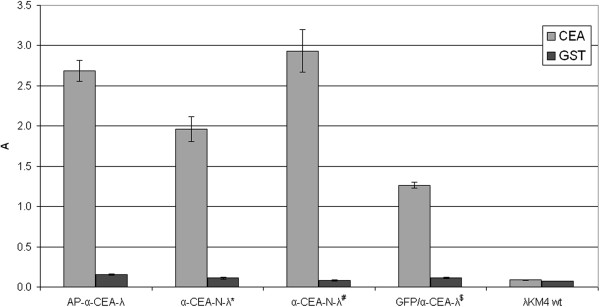
**ELISA reactivity of lambda phage displaying GFP or AP and anti-CEA scFv antibody against CEA.** Lambda phage clones, displaying GFP or alternatively AP as a fusion to the C-terminus of the gpD and anti-CEA scFv antibody as a fusion to the truncated gpV, were tested by ELISA against purified CEA and an irrelevant control protein (GST). Anti-lambda polyclonal rabbit serum was used for detection of the bound phage. Wild type λKM4 was included as a negative control. The phage samples were stored at 4°C for (*) 55-months, (#) 23-months or ($) 9-months. Irrelevant GST protein is included as a negative control. Results are expressed as A=A_450_-A_620_. Data reported are the average values of assays performed in duplicate. Error bars represent the standard deviation of the mean.

Next, we co-displayed the bacterial enzyme alkaline phosphatase (AP) and scFv, on the head and tail of lambda phage particles, respectively, constructing AP/α-CEA-λ phage. The protein fused to gpD consisting of AP without its own leader peptide and having an additional flexible linker (SGGG)_3_S was of 463 aa length. The phages displaying AP were identified by transferring phage plaques on the nitrocellulose filter and developing them directly with AP chromogenic substrate (Figure 12). The phage encoding AP fused to gpD and α-CEA scFv fused to gpV formed plaques having AP enzymatic activity and contained the phage reactive with CEA protein (Figure 13). In the last test we used as positive controls several samples of α-CEA displaying phages prepared earlier and conserved at 4°C for a long time (9 to 55 months). It is interesting that also under very long storage the lambda-displayed anti-CEA scFv antibody maintained its reactivity.

**Figure 14:**
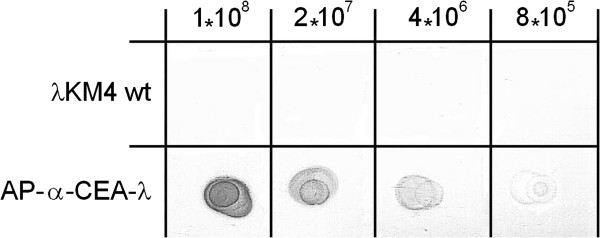
**Dot blot assay of AP/α-CEA-λ phage.** A 1 μl spot of each of the 4 dilutions of the wild type λKM4 and AP-α-CEA-λ phage displaying AP as a fusion to the C-terminus of the gpD and anti-CEA scFv antibody as a fusion to the C-terminus of the truncated gpV, was applied onto the marked squares of a nitrocellulose filter. Then the filter was air dried approximately for 5 min and developed by incubating directly with the AP chromogenic substrate. Phage type is indicated on the left side, phage quantity in spot on the top of the figure.

**Figure 15:**
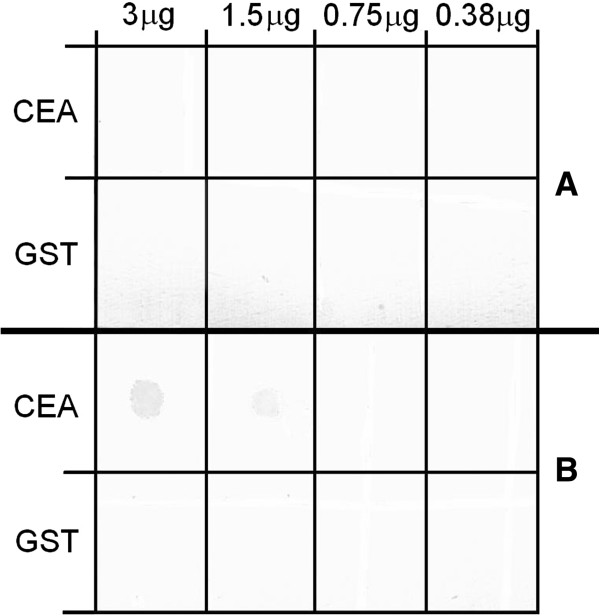
**Detection of the CEA protein by using AP-α-CEA-λ phage.** Four 1 μl drops containing the indicated amount of the purified CEA or irrelevant GST protein were applied onto the marked squares of each nitrocellulose filter. Then the filters were air dried approximately for 5 min and incubated with 10^10^ PFU/ml phage solutions for 1 h at RT, washed and developed by using AP chromogenic substrate. Filter **A** was incubated with the wild type phage λKM4, filter **B** with the AP-α-CEA-λ phage displaying AP as a fusion to the C-terminus of the gpD and anti-CEA scFv antibody as a fusion to the C-terminal end of the truncated gpV.

**Figure 16:**
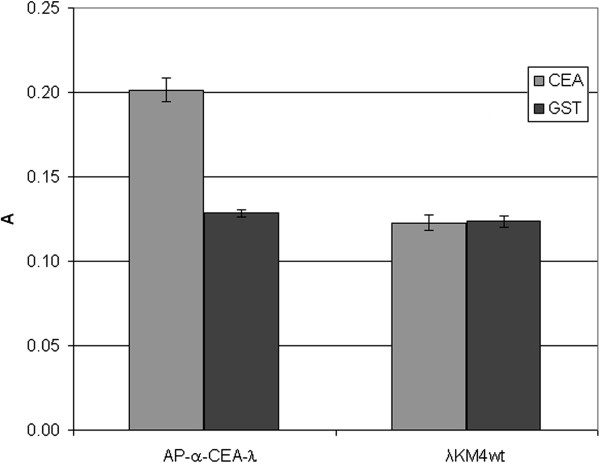
**Detection of CEA protein in ELISA applying AP/α-CEA-λ phage.** AP/α-CEA-λ phage, displaying AP as a fusion to the C-terminus of gpD and scFv anti-CEA antibody to C-terminus of the truncated gpV, was tested by ELISA against purified CEA and an irrelevant GST protein, and developed directly by adding an AP chromogenic substrate. λKM4 wild type phage was also tested as a negative control. Results are expressed as A=A_405_-A_620_. Data reported are the average values of assays performed in duplicate. Error bars represent the standard deviation of the mean.

We used dot blot assay to determine sensitivity of the detection system based on the AP-displaying bacteriophage (Figure 14). According to the test, about 10^7^ PFU were easily detectable by developing with AP chromogenic substrate. Detection of CEA protein first blotted on the membrane and then incubated with the AP/α-CEA-λ phage with following substrate developing demonstrated that the phage works like a specific anti-CEA antibody conjugated with AP (Figure 15), but it is easier to produce through the bacteria infection and propagation. We tested AP/α-CEA-λ phage in direct ELISA, that means by developing a positive signal by adding AP substrate directly after incubation with AP/α-CEA-λ phage. We have got positive result also in this case (Figure 16).

### Display efficiency in gpV system

**Figure 17:**
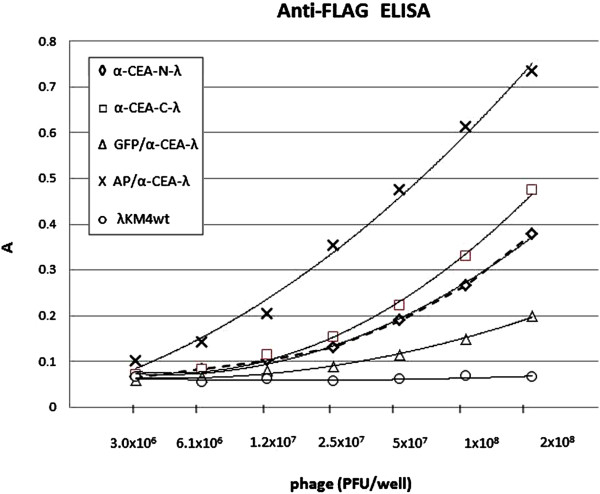
**Efficiency of scFv display on the phage capsid.** Lambda phage clones, displaying scFv at the N-terminus of gpD (α-CEA-N-λ), at the C-terminus of gpD (α-CEA-C-λ), or double displaying scFv fused to truncated gpV and GFP at the C-terminus of gpD (GFP/α-CEA-λ) and double displaying scFv fused to truncated gpV and AP at the C-terminus of gpD (AP/α-CEA-λ), were evaluated for the scFv/FLAG display efficiency. On the abscissa are indicated the amount of wild type phage measured in pfu. The amounts of the phage used in ELISA have been normalized relative to wild type phage in order to have identical number of physical particles per well. ELISA was performed by using anti-lambda antibody-coated plates and the displayed scFv co-expressed with FLAG peptyide was revealed by using an anti-FLAG AP-conjugated antibody. Results are expressed as A=A_405_-A_620_.

We compared display efficiency of scFv antibodies in gpD and gpV-fused phages. The phage particles of anti-CEA-N-λ, anti-CEA-C-λ, GFP/α-CEA-λ and AP/α-CEA-λ were purified. The amounts of the phages were normalized relative to wild type phage and then the phages were analysed in ELISA for quantification of scFv antibody fragment expressed together with FLAG peptide in recombinant phages, by using anti-lambda coated plates and an anti-FLAG AP-conjugated antibody (Figure 17). Based on the known incorporation levels of scFv antibody fused to gpD in anti-CEA-N-λ (about 50%, that corresponds to 210 recombinant molecules of 420 total gpD molecules/phage particle) and in anti-CEA-C-λ (up to 90%, 378 recombinant molecules/phage particle) [10] and finding that the scFv/FLAG display efficiency in GFP/α-CEA-λ is 2.3 times lower than in anti-CEA-N-λ phage and 3 times lower than in anti-CEA-C-λ, we can roughly estimated the incorporation level of gpV protein fused to anti-CEA scFv antibody is 91 to 126 molecules per phage particle that is about half of total gpV. A particularly high signal of AP/α-CEA-λ is explained by contribution of AP, displayed directly on the phage capsid, in ELISA developed with the AP-conjugated anti-FLAG antibody.

## Discussion

In this work we explored (a) the possibility to display GFP on the capsid of the bacteriophage lambda as fusion to the head protein gpD by inserting GFP into different positions of the protein gpD, and (b) the possibility of simultaneous display of two different large proteins (GFP/scFv or AP/scFv) on the capsid using both the head- and the tail-based display platforms.

First, we efficiently displayed the functional GFP by fusion to the N- or C-terminus of the lambda protein D by cloning GFP gene in the vectors λKM8 and λKM10, respectively. However, in the case of C-terminal fusion an amber codon was introduced between gpD and GFP gene for the conditional expression of the GFP in host suppressor bacteria, thus, producing both fused and wild type gpD. This procedure was done because fusion of large proteins to the C-terminus of gpD disturbs phage assembly and results in poor productivity of the phage, forming small plaques, and, therefore, to rapid accumulation of phage revertants with growth advantage [10]. Both phages GFP-N-λ and GFP-C-λ formed normal size plaques as compare to the wild-type λKM10 vector phage and stably expressed GFP even after several consecutive amplification cycles. We noted a lower incorporation level of GFP in the lambda head as compared to scFv described earlier [10], even if both proteins have similar size. We suppose the spatial constrains could influence incorporation of recombinant protein in the phage capsid. Probably scFv is more compatible to phage assembly then GFP.

Considering that both the efficacy of assembly of a recombinant phage and the amount of the foreign protein exposed on the capsid depend on the insertion site, we tried to identify new possible fusion positions inside of the gpD protein suitable for phage display. This idea was supported by cryoelectron microscopy obtained by Yang and colleagues [24] who revealed the orientation of gpD trimers in the phage particle and showed that the positions of both termini of gpD reside on the side of the trimer that binds capsid. Even if both N-terminal and C-terminal fusions have been successfully displayed on the lambda capsid earlier [13,15,16,25-29] we wondered whether it is possible to insert a large protein like GFP in gpD, in sites exposed outside and, thus, more suitable for protein display. The three new possible insertion sites in gpD were chosen outside the hydrophobic core on the bottom side of the gpD trimer, between two successive β-strands and among the hydrophilic amino acids (Ser^42^-Ser^43^, Tre^52^-Tre^53^, Tre^95^-Lys^96^), in order to better expose the foreign protein to the solvent. According to our data, the C-terminus of gpD was found to be the best position for fusion of recombinant proteins, in terms of display efficiency, phage particle production and stability. Conditional expression of the fusion GFP at the C-terminal of gpD improved particle production and phage stability as compared to expression of large protein domains at the C-terminus without amber codon.

The next part of our work consisted in the simultaneous display of two foreign proteins on the lambda capsid. The idea to construct a phage of double specificity, able to target a receptor of interest and at the same time capable to capture an effector molecule, is not new, but very attractive because such system can easily be adapted to various biological systems avoiding complex chemical conjugations. However, bifunctional phages have poorly been investigated even in the well-studied filamentous phage system [33-36]. In fact, only a single report published, according to our knowledge, describes the construction of a lambda phage that simultaneously displays an ubiquitinylation motif (10-aa peptide) fused to the C-terminus of gpD and a CD40 binding motif (12-aa peptide) fused to the tail protein gpV [20]. With the aim to develop lambda-based gene-transfer vector the authors suggested that ubiquitinylation of the capsid and proteasomal degradation would trigger lambda head uncoating and improve phage-mediated gene transfer, while CD40-binding peptide would stimulate the binding of phage particles to the receptor of mammalian cells with following internalization. The new bacteriophage lambda vector simultaneously displaying both peptides demonstrated an enhanced capacity of phage-mediated gene transfer of a luciferase reporter gene in CD40-positive mammalian cells as compared to unmodified phage, or phage displaying only one of the peptides, or a mixture of phages displaying the two peptides separately. This study showed a potential utility of bifunctional lambda phages, but did not investigate the capacity of the lambda vector for double display of large proteins.

In the present work we demonstrate that large protein domains can not be displayed simultaneously on the same molecule of gpD protein, probably because steric constrains disturb assembly of the recombinant phage. Accumulated unassembled protein probably has toxic effect on the infected bacterial cells and stimulates fast selection of revertant phages losing the inserted genes. However, we can not exclude that the gpD-based double display system would tolerate a mosaic capsid where different molecules of gpD are modified at the N- or C-terminal side separately.

Regarding to the double display based on the gpD and gpV display platforms, we cloned successfully the GFP (238 aa) or the AP (449 aa) at the C-terminus of gpD and scFv anti-CEA (242 aa) at C-terminus of a truncated version of gpV. Mosaic phage capsids were composed of wild type proteins gpD and gpV and modified subunits. The GFP/α-CEA-λ phage formed fluorescent plaques with a brightness comparable to the GFP-C-λ, displaying the GFP alone. Good recognition of the CEA protein by GFP/α-CEA-λ phage in ELISA demonstrated the possibility of double displaying functional large proteins on the lambda phage. However, the ratio between particle number and PFU in the sample of the phage GFP/α-CEA-λ was found to be 10 times higher as compared to wt phage. This means that the infectivity of the GFP/α-CEA-λ is disturbed when the lambda tail is involved in the display of large proteins. Detailed analysis of the incorporation level of GFP revealed that it is also slightly lower in the double displaying phage GFP/α-CEA-λ than in GFP-C-λ.

We immunostained *in vitro* CEA-expressing LoVo cells by using GFP/α-CEA-λ phage and found that this phage is able to target GFP to CEA positive tumor cells as compared to the control phage expressing GFP alone. Moreover, phage targeting was efficient also *in vivo* in the mice grafted with HT29 and LoVo CEA-expressing tumors. The phage was detectable in the tumor as infective phage particles at least up to 72 h after i.v. injection. Considering that titration of the infective particles might not reflect properly and quantitatively the phage presence in different organs, because of the likelihood of proteolysis in tissues and possible loss of infectivity, we confirmed phage localization in the tumor by the tissue staining.

Regarding to AP/α-CEA-λ phage, we were able to stain CEA protein directly in immunoblotting experiment by using this double displaying phage. Additionally, the AP/α-CEA-λ was reactive to give a positive signal in direct ELISA. This result was possible even if AP is twice the size of GFP (463 aa vs 238 aa) and theoretically should impair more the phage capsid assembly.

In the present work we observed relatively high incorporation level of scFv fused to gpV protein in the phage tail as compared to previously reported data of Maruyama [30]. Upon partial suppression of amber mutation introduced in native *V* gene, Maruyama and co-authors co-expressed truncated gpV and truncated gpV fusion and observed 0 to 3 recombinant molecules incorporated in the phage particle. At least five copies of α-peptide per phage tail were incorporated in the phage bearing a normal copy of the V gene and grown on host strain expressing α-peptide as a C-terminal fusion to full-length gpV [37]. In our two-gene based vector system, λKM10 [17], the recombinant bacteriophages AP/α-CEA-λ or GFP/α-CEA-λ have mosaic structure of their tails, composed of native gpV and truncated gpV fused to scFv gene. In this case about half of total gpV incorporated in the phage tail are fused to anti-CEA scFv antibody.

Bifunctional phage nanoparticles displaying an antibody fragment and a reported/effector moiety are potentially useful for tumor cell targeting and delivery of diagnostic or therapeutics. The phages are commonly recognized as safe for humans, because they are not able to infect mammalian cells [38]. In fact, the coliphage ϕX174 has been used for decades as a standard antigen for the evaluation of immunity in clinical medicine [39]. Moreover, the lambda phage is known to be a highly stable. Indeed, in this study we tested lambda displaying scFv anti-CEA antibody, that was still active after 55-month storage at the 4°C.

## Conclusions

Targeted nanoparticles have been engineered to display an anti-CEA single-chain antibody fragment and green fluorescent protein or bacterial alkaline phosphatase simultaneously by modifying both the gpV, the protein forming the phage tail, and gpD, the head-stabilizing protein of bacteriophage lambda. The present data demonstrate the feasibility and potential usefulness of the lambda dual display system for possible use in biomedical applications.

## Abbreviations

Aa: Amino acid; AP: Alkaline phosphatase; CEA: Carcino embryonic antigen; ELISA: Enzyme-linked immunosorbent assay; GFP: Green fluorescent protein; GST: Glutathione S-transferase; HRP: Horseradish peroxidase; i.p.: Intraperitoneal; i.v.: Intravenous; PBS: Phosphate-buffered saline; PEG: Polyethylene glycol; PFU: Plaque-forming unit(s); scFv: Single-chain variable antibody fragment; SDS/PAGE: Sodium dodecyl sulphate/polyacrylamide gel electrophoresis; wt: Wild type.

## Competing interests

The authors declare that they have no competing interests.

## Authors’ contributions

EP carried out the cloning experiments, performed preparation, purification and characterization of the phage clones, and drafted the manuscript. PV participated in the phage targeting *in vivo*, performed phage preparation and participated in the manuscript editing. VDA contributed to the experiments *in vivo*. RDS participated in the design of the study and the manuscript editing. OM participated in the lambda clones construction and characterization, coordinated research and prepared the manuscript. All authors read and approved the final manuscript.
